# Temperature-Frequency Converter Using a Liquid Crystal Cell as a Sensing Element

**DOI:** 10.3390/s120303204

**Published:** 2012-03-07

**Authors:** Carlos Marcos, José M. Sánchez Pena, Juan C. Torres, José Isidro Santos

**Affiliations:** Grupo de Displays y Aplicaciones Fotónicas, Departamento de Tecnología Electrónica, Universidad Carlos III de Madrid, Avda. de la Universidad s/n, E28911, Leganés, Madrid, Spain; E-Mails: jmpena@ing.uc3m.es (J.M.S.P.); jctzafra@ing.uc3m.es (J.C.T.); jsantos@ing.uc3m.es (J.I.S.)

**Keywords:** temperature sensor, multivibrator circuit, nematic liquid crystal

## Abstract

A new temperature-frequency converter based on the variation of the dielectric permittivity of the Liquid Crystal (LC) material with temperature has been demonstrated. Unlike other temperature sensors based on liquid crystal processing optical signals for determining the temperature, this work presents a system that is able to sense temperature by using only electrical signals. The variation of the dielectric permittivity with temperature is used to modify the capacitance of a plain capacitor using a LC material as non-ideal dielectric. An electric oscillator with an output frequency depending on variable capacitance made of a twisted-nematic (TN) liquid crystal (LC) cell has been built. The output frequency is related to the temperature of LC cell through the equations associated to the oscillator circuit. The experimental results show excellent temperature sensitivity, with a variation of 0.40% of the initial frequency per degree Celsius in the temperature range from −6 °C to 110 °C.

## 1. Introduction

Flat panel displays for electro-optical visual applications have been the core market of LC for the last 40 years. However, in the last decade new LC-based applications are focusing the interest of the research community [1,2]. New LC materials with *ad-hoc* anisotropic properties have been synthesized and innovative non-optic applications such as passive tunable radio frequency (RF) and microwave devices have been fabricated and reported [3–6].

Concerning sensor applications, different types of LC-based sensors such as biological, shear force or pressure have been previously developed [7–10]. Because of the dependence of optical properties on temperature, measuring devices based on LC materials have been widely reported as either optical or electrooptical temperature sensors [11–13]. This research presents a novel temperature-frequency (T-f) converter based on an LC cell which behaves as pure electric transducer.

**Figure 1.**
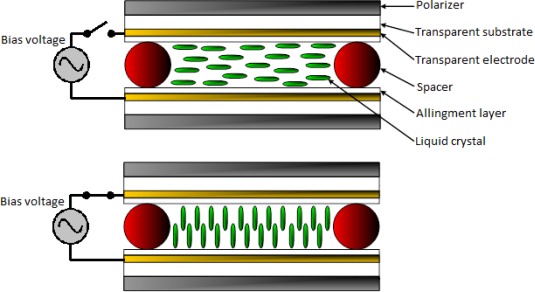
Structure and working principle of a homogeneously aligned nematic liquid crystal cell.

Basically, the structure of a LC cell consists of two parallel transparent plates or substrates with a conductive layer (electrode) in their inner surfaces. Inner surfaces are conditioned by a specific alignment process to achieve a homogenous molecular order of the LC material which is retained between the parallel plates. The nematic phase is one of the most widely used LC phase for a variety of electro-optic applications. The orientation of the molecules in a nematic phase substance can be altered by the application of an external electric field. In order to build practical devices, liquid crystals are sandwiched between two electrodes; therefore, the molecules tilt angle can be driven by an external electric field applied between both electrodes (Figure 1).

Due to the anisotropic electric properties of the LC materials, the dielectric permittivity depends on their molecules orientation; when no electric field is applied to the LC cell, the molecules will remain parallel to the substrates, thus the perpendicular dielectric permittivity is measured, ɛ_⊥_. However, if a strong electric field is applied, the molecules tilt to the vertical position and the director points in the electric field direction so, in this case, the parallel dielectric permittivity is measured, ɛ_||_. Any electric field with intermediate values can provide a dielectric permittivity between ɛ_⊥_ and ɛ_||_ of the LC material.

**Figure 2.**
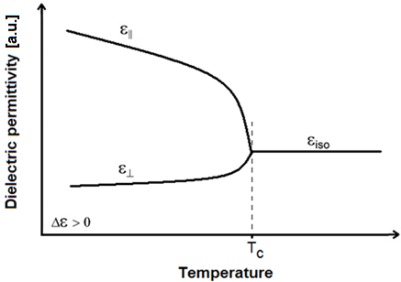
Dependence of dielectric permittivity for a typical nematic liquid crystal on temperature.

As previously mentioned, nematics are the commonly used LC phase and they usually have positive dielectric anisotropy, this means that parallel dielectric permittivity is greater than perpendicular (ɛ_||_ > ɛ_⊥_). The dielectric permittivities depend on temperature and, for positive LC materials, this dependence is stronger for the parallel constant than for the perpendicular one [14]; Figure 2 shows the dielectric permittivity dependence on temperature for a typical nematic LC (NLC) material. As can be seen in Figure 2, bellow the clearing temperature (T_C_) the parallel permittivity slope is sharper than the perpendicular one; the research presented in this work is based on the influence of temperature in positive NLC permittivity. When temperature exceeds the clearing point the LC transforms into an isotropic liquid and the NLC permittivity remains nearly constant

An LC cell can be electrically modeled by an equivalent passive circuit; for intermediate frequencies of the applied bias voltage (from 1 Hz to 10^4^ Hz), the electrical behavior of the LC cell is a parallel circuit of a resistor and a capacitor [15]. The resistance and capacitance values depend on the cell dimensions and the LC material. The resistance value is high and approximately constant but, because of the LC dielectric anisotropy, the capacitance value will depend on the applied electric field, which modifies the LC molecules tilt angle, as well as on the environmental temperature.

According with the LC electric equivalent circuit and the LC permittivity behavior with temperature, an LC cell can be modeled as an RC parallel circuit with a capacitance value depending not only on the electric applied field, but also on the environmental temperature as shown in Figure 2.

In this work, a new temperature-sensing electric system based on the dependence of an NLC cell capacitance with the temperature is presented. The system consists of a square wave generator (SWG) circuit where an NLC cell is used as the temperature dependent capacitance, which controls the generator output frequency. Hence, a temperature variation will change the square wave frequency of the electric oscillator. The developed temperature-frequency (T-f) converter is based on a simple electronic circuit, which provides a wide temperature sensing range, high sensitivity and good stability.

## 2. System Design and Working Principle

**Figure 3.**
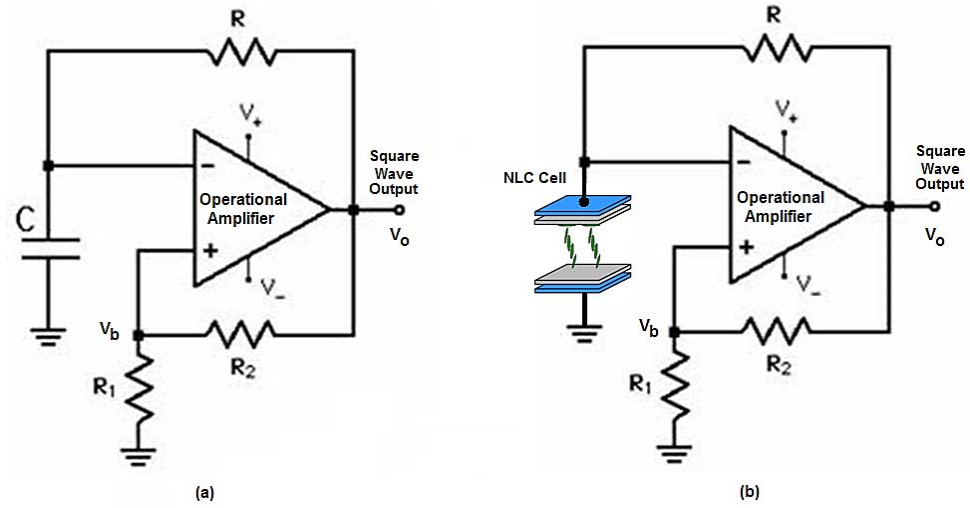
(**a**) Multivibrator oscillator circuit. (**b**) Multivibrator circuit with NLC cell.

The T-f converter is based on the multivibrator circuit shown in Figure 3(a). The multivibrator circuit is able to generate a square wave output signal, which switches between two symmetrical levels, the high level with a voltage of *+V_o_* and the low level of *−V_o_*. The output square wave is a 50% duty cycle signal, therefore the output signal maintains the high level during 50% of the period and the remaining time output level is low.

The multivibrator circuit is implemented with one operational amplifier, three resistors (*R, R_1_*, and *R_2_*) and one capacitor (*C*). The period of the square wave output signal is two times higher than the capacitor charging time from *–V_b_* to *+V_b_* voltages, where *V_b_* is the feedback voltage ratio given by *R_1_* and *R_2_*; the charging time is controlled by the *RC* time constant. Therefore, the period time *T* is given by the equation:

(1) $$T=2\cdot R\cdot C\cdot \text{ln}\left(\frac{V_{0}-V_{b}}{V_{0}+V_{b}}\right)$$

where the feedback voltage ratio, *V_b_*, is constant and can be mathematically expressed in the form:

(2) $$V_{b}=V_{0}\cdot \frac{R_{1}}{R_{1}+R_{2}}$$

and period time equation becomes:

(3) $$T=2\cdot R\cdot C\cdot \text{ln}\left(\frac{{2R}_{1}+R_{2}}{R_{2}}\right)$$

Therefore, the period time, *T*, only depends on the value of the passive components (resistors and the capacitor).

**Figure 4.**
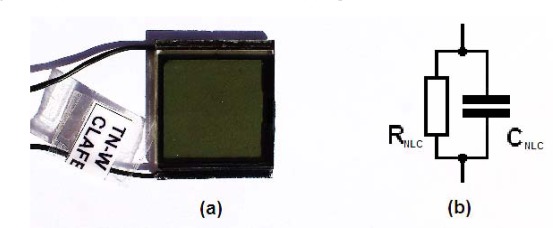
(**a**) NLC cell used in the T-f converter. (**b**) Equivalent electric circuit of the NLC cell.

The T-f converter is obtained by a simple modification of the multivibrator circuit. When the capacitor is replaced by an NLC cell (Figure 3(b)), the frequency only depends on the NLC cell temperature because the average electric field applied to the NLC cell (V_b_) is constant. Commercial off the shelf twisted nematic liquid crystal cell manufactured by LC-Tec Company [16] with an effective area of 21 × 21 mm^2^ was used in the experiments (Figure 4(a)). As is mentioned above, an NLC cell under an AC bias voltage can be electrically modeled as an R-C parallel circuit (Figure 4(b)). This equivalent electrical circuit is valid for an applied bias voltage with frequencies in the range from 1 Hz to 10^4^ Hz. Therefore, to determine the capacitance and resistance values, the NLC cell is electrically analyzed applying a bias voltage of 5 kHz and a square signal with a 50% duty cycle. This bias voltage is equivalent to the electrical conditions in the multivibrator circuit.

**Figure 5.**
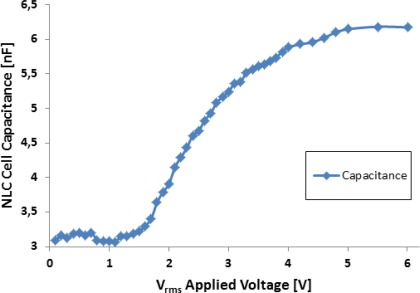
Equivalent capacitance of the NLC cell used in the T-f converter.

The LC device was electrically characterized by using the experimental procedure described in [5] that is able to derive the capacitance value of the LC device as a function of the applied voltage. Figure 5 shows the experimental capacitance value obtained for the NLC cell applying different voltage levels at 5 kHz. As expected, due to the LC molecular orientation with the electrical field, the equivalent capacitance presents a high dependency on the applied voltage. The measured value of the capacitance ranges from 3 nF (when no voltage is applied and the molecules remain parallel to the substrates) to 6 nF (for applied voltages above 5 Vrms that keeps the molecules perpendicular to the substrates). However, the equivalent resistor parameter is almost constant with an experimental obtained value of 1.5 MΩ.

With the obtained experimental values for the electric equivalent parameters of the NLC cell, the impedance can be mathematically expressed as follows:

(4) ZNLC=RNLC⋅1j2πf CNLCRNLC+1j2πf CNLC

where *C_NLC_* and *R_NLC_* are the capacitance and resistance values of the NLC cell and *f* is the frequency of the applied signal to the NLC cell. Expanding the Equation (4), the impedance is given by:

(5) $$Z_{\mathrm{NLC}}=\frac{R_{\mathrm{NLC}}}{1+{\left(2\pi f\;C_{\mathrm{NLC}}R_{\mathrm{NLC}}\right)}^{2}}+\frac{2\pi f\;C_{\mathrm{NLC}}R_{\mathrm{NLC}}{}^{2}}{j[1+{\left(2\pi f\;C_{\mathrm{NLC}}R_{\mathrm{NLC}}\right)}^{2}]}$$

Considering the experimental values obtained for *C_NLC_* (3 nF ≤ *C_NLC_* ≤ 6 nF) and *R_NLC_* ≈ 1.5 MΩ and a working frequency of around 5 kHz, the Equation (5) can be simplified in the following way:

(6) $$Z_{\mathrm{NLC}}\approx \frac{1}{j2\pi f\;C_{\mathrm{NLC}}}$$

Additionally, it can be derived from Equation (6) that the magnitude of the impedance associated to the equivalent resistance of the NLC cell is higher compared to the magnitude of the impedance associated to the equivalent capacitance. Consequently, the electrical equivalent circuit of the NLC cell can be assumed as a pure capacitor in the SWG multivibrator circuit.

## 3. Experimental Setup

**Figure 6.**
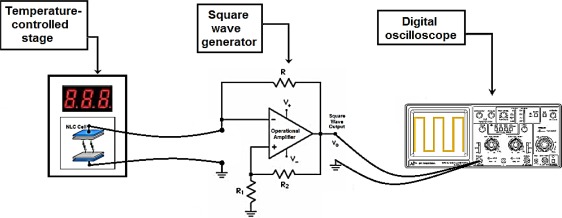
Experimental setup for the temperature-frequency converter.

The temperature-frequency converter has been implemented based on Figure 3(b) as a multivibrator circuit. A TL081 general purpose operational amplifier has been used. The feedback resistors, R_1_ and R_2_, and the charging resistor, R, take appropriate values to set an output signal frequency of 5 kHz. With this working frequency, the electric behavior of the NLC cell can be assumed as a pure capacitance. The experimental setup implemented for the NLC SWG temperature sensor is shown in Figure 6.

The NLC cell used as a sensor element was placed inside a Linkam LTS350 temperature-controlled stage for accurate temperature measurements. This system was used as a temperature reference to calibrate the T-f converter implemented. The multivibrator circuit was outside the isothermal stage, thus, the sensor element is the unique circuit element affected by the temperature variations. Two electrical wires were used to connect the NLC cell with the circuit. The output square signal was acquired using a digital oscilloscope to measure the output signal frequency and derive the corresponding temperature values.

## 3. Experimental Results and Discussion

**Figure 7.**
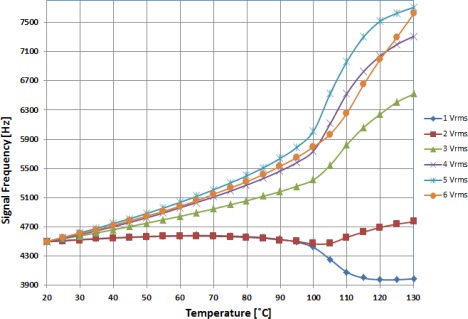
Variation of output frequency as a function of the temperature for different voltages applied to NLC cell.

The results obtained from the experimental setup are shown in Figure 7. Using the isothermal stage, the temperature was linearly increased from room temperature (20 °C) to nearly above the NLC clearing point. For this temperature range, different applied voltages to the NLC cell were tested in order to obtain the optimal value in terms of the sensitivity and linearity of the system. A starting frequency of 4.5 kHz was set at room temperature in all tests carried out. Results show the output signal frequency is a function of the temperature. As has been expected, as a consequence of parallel permittivity (ɛ_||_) highest dependence on temperature, the frequency variation is greater when higher average voltage is applied to the NLC cell. Figure 7 shows a sharp change of the curve slopes around a temperature of 100 °C. This is due to the NLC cell getting close to its clearing temperature.

When high voltage is applied to the NLC cell, molecules are reoriented along the electric field and perpendicular to the substrates. At this high voltage regime slight voltage variations have no consequence in molecular order, consequently similar curve shapes should be expected for voltages above 4 Vrms in Figure 7. However, though the molecular order is the same, the curve of permittivity dependence on temperature is affected by the high voltage applied to the LC cell; therefore curve shapes at high voltage regime are different [17].

**Figure 8.**
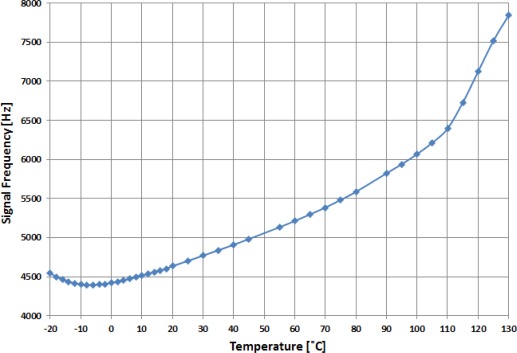
Output frequency variation as a function of temperature for an NLC cell (applied voltage of 6 V_rms_).

**Figure 9.**
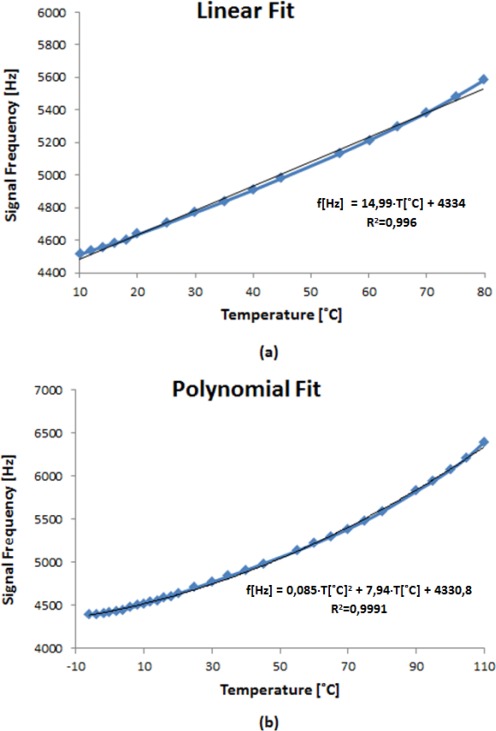
(**a**) T-f response curve with a linear fit. (**b**) T-f response curve with a second order polynomial fit.

From the experimental results, the optimum value chosen for the applied voltage to the LC cell is 6 V_rms_ due to a better linearity compared to other values. Once this voltage is fixed, the temperature range has been extended to check the operating limits of the implemented system. Figure 8 shows the output signal frequency of the oscillator circuit as a function of temperature. The measurements were experimentally obtained in the temperature range from −20 °C to 130 °C. The sensor response can be linearly fit in the temperature range from 0 to 80 °C with a sensitivity of 14.37 Hz/°C (Figure 9(a)).

However, in the temperature range from −6 to 110 °C, the T-f converter response can be fitted by using a second order polynomial function. Now, the system exhibits a lower sensitivity at low rather than at high temperatures where the sensitivity achieves 25.2 Hz/°C (Figure 9(b)). The results demonstrate the right operation of a T-f converter developed in a wide temperature range using an NLC cell working as an electric transducer.

## 4. Conclusions

In this work, a new type of temperature-frequency converter based on a SWG circuit using an NLC cell behaving as an electric transducer has been proposed and developed. Unlike previously reported temperature sensors based on LC materials that need optical signals for temperature sensing, the presented system only uses electric signal to measure temperature. This work provides a new approach in the field of temperature sensing, with significant potential in applications such as *in situ* temperature sensing in embedded LC display projectors, where temperature-related malfunctions are common.

Temperature dependence of the NLC dielectric permittivity causes a frequency variation in the oscillator output signal. Different sensor sensitivities can be achieved by adjusting the control voltage applied on the NLC cell. The experimental results show that the T-f converter response can be fitted using a second order polynomial over a wide temperature range from −6 to 110 °C. In this temperature range, the T-f converter shows good sensitivity, stability and a negligible hysteresis.

Unlike other temperature optical sensors based on liquid crystals, the system implemented has a wider temperature sensing range. Moreover, the simple construction of this sensor shows an advantage in practical applications allowing its integration in more complex instrumentation systems

Future studies will further investigate the use of *ad-hoc* manufactured LC cells in the T-f converters in order to optimize parameters such as linearity, repeatability, sensing range, among others. Additionally, the use of an NLC device with positive permittivity and homeotropic alignment would cancel the voltage effect in this kind of devices
